# Vitamin D and Insulin-Dependent Diabetes: A Systematic Review of Clinical Trials

**DOI:** 10.3390/nu16071042

**Published:** 2024-04-03

**Authors:** Yuval Dadon, Lior Hecht Sagie, Francis B. Mimouni, Iris Arad, Joseph Mendlovic

**Affiliations:** 1 Ministry of Health, Jerusalem 9101002, Israelsefi.mendlovic@moh.gov.il (J.M.); 2 Leumit Health Services Research Center, Tel Aviv 6473817, Israel; fbmimouni@gmail.com; 3 Sackler School of Medicine, Tel Aviv University, Tel Aviv 6997801, Israel; 4 School of Medicine, Hebrew University of Jerusalem, Jerusalem 9190500, Israel; 5 Shaare Zedek Medical Center, Affiliated with the Hadassah-Hebrew University School of Medicine, Jerusalem 9103102, Israel

**Keywords:** vitamin D, vitamin D metabolites, insulin-dependent diabetes mellitus, type 1 diabetes (T1DM), beta-cell preservation, glycemic control

## Abstract

(1) Background: Vitamin D supplementation after type 1 diabetes mellitus (T1DM) onset has led to conflicting results on beta-cell preservation. Aim: This paper presents a systematic review to verify whether randomized prospective controlled trials (RCTs) demonstrate that improved vitamin D status confers protection on T1DM. (2) Methods: A systematic review was conducted up until 18 January 2024 according to Preferred Reporting Items for Systematic Reviews and Meta-Analyses (PRISMA) guidelines, searching MEDLINE, MEDLINE In-Process, Embase, Cochrane Database of Systematic Reviews, and Cochrane Central Register of Controlled Trials, using keywords “vitamin D”, “type 1 diabetes”, and “children”. (3) Results: Following the above-mentioned search process, 408 articles in PubMed and 791 in Embase met inclusion criteria. After removing duplicates, 471 articles remained. After exclusion criteria, 11 RCTs remained. Because of major heterogeneity in design and outcomes, no meta-analyses were conducted, allowing only for qualitative analyses. There was no strong evidence that vitamin D supplementation has lasting effects on beta-cell preservation or glycemic control in new-onset T1DM. (4) Conclusions: More rigorous, larger studies are needed to demonstrate whether vitamin D improves beta-cell preservation or glycemic control in new-onset T1DM. Because T1DM may cause osteopenia, it is advisable that patients with new onset T1DM have adequate vitamin D stores.

## 1. Introduction

Vitamin D has major immunomodulatory effects that are the focus of much research. Animal and in vitro studies are suggestive of a functional role of vitamin D3 as an immune modulator. Such effects of vitamin D might be highly relevant in immunologic diseases such as type 1 diabetes mellitus (T1DM). Indeed, vitamin D has been shown to inhibit the synthesis of pro-inflammatory cytokines, affects adaptive Th1 responses [1], and promotes a tolerogenic phenotype in human dendritic cells, which induces Foxp3+ regulatory T cells [2]. Many tissues, including activated T and B human lymphocytes, express receptors for the active form of vitamin D3 [3]. In animal studies, cholecalciferol (or vitamin D3) suppresses the proliferation of lymphocytes and modifies the helper T-cell subtypes 1 and 2 (TH1/TH2) cytokines profile [4]. It is known that TH1 cytokine is associated with cell-mediated immunity, while TH2 cytokine plays an important role in humoral immune responses [5]. Type 1 diabetes is believed to be triggered by TH1 cells, while TH2 cells may be protective [6]. Exposure of human beta cells in vitro to 1,25(OH)2D3 (calcitriol) may protect them from death [7]. One study showed that vitamin D supplementation in pregnancy and during infancy may be associated with a decrease in the risk of T1DM [8]. Supplementation with 1,25(OH)2D3 after T1DM onset has led to conflicting results on the preservation of beta-cell function [9,10,11]. 

Thus, we undertook this systematic literature review to establish whether prospective randomized controlled clinical trials (RCTs) show that improved vitamin D status confers protection on T1DM. 

## 2. Methods

### 2.1. Search Strategy

This systematic review followed the guidelines of PRISMA (Preferred Reporting Items for Systematic Reviews and Meta-Analyses). We searched MEDLINE, MEDLINE In-Process, Embase, the Cochrane Database of Systematic Reviews, and the Cochrane Central Register of Controlled Trials up until 18 January 2024. A structured search strategy was conducted using high frequency keywords and a thesaurus explode operation aimed to optimize recall. Search filters were used to improve precision [12]. We performed the search using the term “vitamin D” and the second one using the terms “vitamin D”, “type 1 diabetes”, and “children”. The filters consisted of age, the type of documents (randomized controlled trial or systematic review), and language (English only). Reference lists of all selected papers were scrutinized with the aim of finding references that the computerized search did not find. All clinical trials of vitamin D (D3 or D2 and their metabolites) were retained for full-text review. Interventions eligible for assessment were those involving any dose of vitamin D supplementation. The intervention could be compared to placebo or no intervention, or to other doses of vitamin D, or any other treatment. Our main goal was to review every article for new evidence on vitamin D administration or supplementation and how it may affect type 1 diabetes. The search included systematic reviews, meta-analyses, clinical trials, and guidelines related to vitamin D and type 1 diabetes mellitus. We only included in the analyses trials that included a random allocation. We noted whether or not the study had been performed in children only (≤18 years) or in children and adults. 

### 2.2. Data Collection

Titles and abstracts of records identified by the search were reviewed by three authors (LHS, YD, and FBM) and either “ordered” or “excluded” as full-text articles. Suitability for inclusion was determined based upon preset inclusion and exclusion criteria. A data collection form was used to extract data and record information on the design, methods, and participants from each study. Disagreements were resolved through consensus among reviewers.

## 3. Results

**Figure 1:**
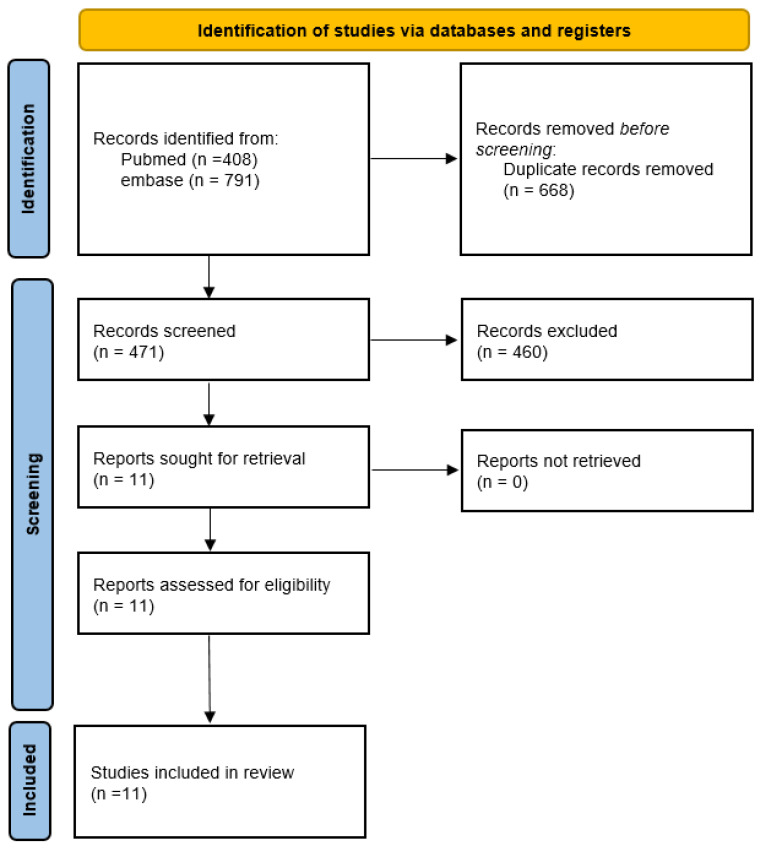
PRISMA 2020 flow diagram of this study.

We retrieved 408 articles from PubMed and 791 articles from Embase. After duplicates removal and exclusion criteria, 11 RCTs remained eligible (see the flow chart in Figure 1) [9,10,11,13,14,15,16,17,18,19,20].

### Types of Intervention

Three studies [9,10,11] used calcitriol for intervention 0.25 mcg/day [10,11] or 0.25 mcg every other day [9]. The latter study [9] used nicotinamide in the control group. Studies 10 and 11 used different time points for outcomes, rendering meta-analyses not possible. Thus, all three studies [9,10,11] were only analyzed qualitatively. 

One study used cholecalciferol at daily 2000–4000 units [18]. Its results could only be analyzed qualitatively. 

In one study, a mega dose of D3 was given, equivalent to approximately 2857 IU/day [17]. This study used a unique crossover design; therefore, it could not be used for meta-analysis [17] and was only analyzed qualitatively. 

One study used a mega dose of ergocalciferol, equivalent to approximate daily dose of 7143 IU, then 3571 IU [20]. This study could only be analyzed qualitatively, similar to another study which used a daily dose of 70 IU/kg weight [16]. 

Study 14 was the only study to use 1 alpha OH cholecalciferol, and it was retained for qualitative analysis. 

In three studies [13,15,19], cholecalciferol was used at 2000 IU/day. However, one study [19] reported no clinical outcomes and only measured IL-4 and IL-1β, which were not measured in the two other studies [13,15]. A comparison of studies 13 and 15 shows that no meta-analyses could be conducted since Gabbay’s study was a randomized, double-blinded, placebo-controlled trial using a daily 2000 IU D3 in the intervention group, while in Haller’s study, the intervention group used the same D3 dose, but combined it with DHA supplementation and after autologous umbilical cord blood infusion, in an open label design. Thus, studies 13, 15, and 19 were only retained for qualitative analyses. 

In summary, for all 11 studies remaining in the analysis, no meta-analyses could be conducted. The characteristics of the studies are summarized in Table 1. 

Table 2 depicts the outcome variables examined in the various 11 studies from a qualitative standpoint.

Pittocco [9] investigated whether calcitriol supplementation (0.25 mcg every other day) in subjects with recent-onset T1DM (within 1 month of diagnosis) protects residual pancreatic β-cell function and improves glycemic control after 1 year of follow-up. This open-label randomized trial included 70 subjects above the age of 5 years with a mean age of 13.6 years (no age range provided). It is unclear how many patients were randomized to the intervention group; the control group received nicotinamide. No significant differences were observed between groups in respect of baseline/stimulated C-peptide or HbA1c one year after diagnosis, but insulin dose at 3 and 6 months was significantly reduced in the calcitriol group. Results of this study could be interpreted in that calcitriol provides a temporary beneficial effect on residual beta-cell function. However, the alternative could be that nicotinamide is detrimental to residual beta-cell function. 

Walter [10] studied whether daily calcitriol supplementation (0.25 mcg/day) in subjects with recent-onset T1DM (within 2 months of diagnosis) is safe, improves beta-cell function and improves glycemic control after 18 months of follow-up. This prospective, randomized, placebo-controlled clinical trial included 18 subjects in the placebo group and 20 subjects in the intervention group. Subjects ranged from 18 to 39 years (no average age or age range provided). No significant differences were observed in glycemic control or residual beta-cell function after 18 months. 

Bizzari [11] studied whether daily calcitriol (0.25 mcg/day) in subjects with recent-onset T1DM (within 3 months of diagnosis) is safe and improves beta-cell function and glycemic control after 2 years. This prospective, randomized, placebo-controlled clinical trial included 12 subjects in the placebo group and 15 subjects in the intervention group. Subjects ranged from 11 to 35 years and had a median age of 18 years. No significant differences were observed in glycemic control or in residual beta-cell function after 2 years. 

Gabbay [13] studied the effect of vitamin D3 on cytokine levels, regulatory T cells, and decline in residual beta-cell function after daily 2000 IU cholecalciferol in subjects with recent-onset T1DM (within 6 months of diagnosis after 1.5 years of follow-up). This prospective, randomized, placebo-controlled clinical trial included in its final analyses 18 subjects in the placebo group and 17 subjects in the intervention group. Subjects ranged from 7 to 30 years (no median or mean age provided). No significant differences were observed in HbA1C or insulin dose or in C-peptide. After 12 months, chemokine ligand 2 (monocyte chemoattractant protein 1) was significantly higher; additionally, there was an increase in regulatory T-cell percentage (4.55% ± 1.5% vs. 3.34% ± 1.8%) in cholecalciferol vs. placebo groups. Importantly, 18.7% of patients in the cholecalciferol group and 62.5% in the placebo group progressed to undetectable (0.1 ng/mL) fasting C-peptide concentrations, while stimulated C-peptide reached 6.2% in the cholecalciferol group and 37.5% in the placebo group at 18 months. Thus, cholecalciferol as an adjunct of insulin was deemed to be safe and associated with a protective immunologic effect and slower decline of residual beta-cell function. 

Ataie-Jafari [14] studied whether alfacalcidiol preserves beta-cell function when administered at 0.25 mcg twice daily in subjects with recent-onset T1DM (within 2 months of diagnosis). Outcomes were glycemic control and beta-cell function after 6 months. This prospective, randomized, placebo-controlled clinical trial included 25 subjects in the placebo group and 29 subjects in the intervention group. Subjects ranged from 8 to 15 years, with a mean age of 10.7 years. No significant differences were observed in HbA1C, but daily insulin dose was lower and fasting C-Peptide was higher at 6 months of follow-up in the intervention group. This protective effect of alfacalcidiol was stronger in males. No measures of inflammatory cytokines were conducted. 

Haller [15] studied whether autologous umbilical cord blood (UCB) infusion followed by 1 year of supplementation with daily 2000 IU cholecalciferol and docosahexaenoic acid (DHA) (38 mg/kg) can preserve C-peptide in children with recent-onset type 1 diabetes (average 3.8 months, no upper period was described). Subjects ranged from 1–18 years of age (median age 7 years). This open-label, 2:1 randomized study included 15 subjects with stimulated C-peptide > 0.2 pmol/mL who received either autologous UCB infusion, 1 year of daily oral vitamin D (2000 IU), and DHA and intensive diabetes management (N = 10) or intensive diabetes management alone (N = 5). After 1 year, there were no differences in the rate of C-peptide decline between the groups. CD4/CD8 ratio remained stable in treated subjects but declined in control subjects (*p* < 0.03). No changes were seen in regulatory T cell frequency, total CD4 counts, or autoantibody titers. These results could theoretically be caused by autologous umbilical cord blood (UCB) infusion, cholecalciferol supplementation, DHA supplementation, or any combination of the above. 

Treiber studied cholecalciferol supplementation on T regulatory cells (TREGS), frequencies of important immune cells in peripheral blood, and residual beta-cell function. This randomized, double-blinded, placebo-controlled trial included 30 young patients (>6 years of age, range 9.5 to 17.5 years, median 12.5 years) with recent-onset T1DM (within 3 months of diagnosis) assigned to cholecalciferol (70 IU/kg bodyweight/day) or placebo for 12 months. Suppressive capacity of TREGS increased with cholecalciferol from baseline to 3, 6, and 12 months, and change of suppression capacity from baseline to 12 months was significantly higher with cholecalciferol than placebo. However, the percentage of circulating CD4+CD25hiFoxp3+CD127dim Tregs was not altered. There were no clinical correlations performed in this study. 

Shih [17] studied whether correcting vitamin D deficiency in adolescents with T1DM improves glycemia and reduces inflammatory markers. This randomized, prospective, crossover study included 25 adolescents with recent-onset (less than 1 year) T1DM (aged 13–21 years, median 16.7 years) and vitamin D deficiency. Subjects received cholecalciferol (20,000 IU/week) for 6 months, either immediately or after a 6-month delay. The vitamin D-deficient group, when compared with the sufficient group, had significantly higher IL-6 and similar CRP and TNF-α HbA1c. Insulin dosage, CRP, IL-6, and TNF-α were unaffected by the supplementation. Neither glycemia nor markers of inflammation were affected by vitamin D repletion over 6 months. 

Sharma [18] studied whether vitamin D helps improve glycemic control and maintain residual pancreatic beta-cell function. This double-blinded, randomized controlled trial included 52 children (1–18 years, median 9.3 years) assigned to the intervention group (N = 26) (oral vitamin D therapy once a month for 6 months in addition to insulin) versus insulin alone in the control alfacalcidol group (N = 26). The mean C-peptide concentrations rose significantly in the intervention group as opposed to the standard care group (*p* < 0.05). However, at 6 months follow-up, there were no significant differences in HbA1c or in insulin requirement between groups. 

Kadhim [19] studied whether daily 2000 IU vitamin D3 supplementation affects inflammatory biomarkers in 50 newly diagnosed (disease duration < 1 year) pediatric patients (4–12 years old, average 8.5 years). This open-label 1:1 study included 25 patients treated with daily insulin regimen and 25 patients who supplemented with vitamin D3 in addition to insulin for a 90-day period. There was a significant decline in serum IL-1β after 90 days (*p* = 0.049) in the vitamin D-supplemented group, as well as a significant increase in serum IL-4 after 90 days, together with a significant IL-4 reduction in the insulin-treated group only. No significant association between vitamin D with inflammatory markers was observed. No clinical correlations were performed in this study, either in terms of glycemic control or in terms of preservation of beta-cell function. 

Nwosu [20] studied the effect of adjunctive ergocalciferol on residual β-cell function and partial clinical remission in youth with newly diagnosed type 1 diabetes maintained on a standardized insulin treatment for a 12-month period. This double-blinded, 1:1 randomized placebo-controlled trial included 36 subjects (aged 10 to 21 years, disease duration < 3 months, and stimulated C-peptide > 0.2 nmol/L/0.6 ng/mL). Eighteen subjects were given ergocalciferol (50,000 IU of per week for 2 months, later reduced to once every 2 weeks for 10 months) versus placebo in eighteen other subjects. Outcomes were change in stimulated C-peptide, changes in glycemia, insulin dose–adjusted A1c, and inflammatory markers. Serum 25-hydroxyvitamin D at 6 months (*p* = 0.01) and 9 months (*p* = 0.02) were significantly higher in the ergocalciferol group. This study found no statistically significant difference in overall mean fasting C-peptide concentrations (*p* = 0.54), fasting C-peptide (*p* = 0.72), or stimulated C-peptide (*p* = 0.08) between groups.

## 4. Discussion

This systematic review highlights the limitations of the prospective trials that have been published to date on the topic of a potential protective effect of vitamin D supplementation on recent-onset T1DM. 

The heterogeneity of the articles retrieved was considerable to the point that no meta-analyses could be conducted on any of the outcome variables considered. Not all studies were placebo controlled, and 5 studies out of 11 were open-label studies. The control group used nicotinamide instead of placebo in one study, adding another confounder, or autologous blood transfusion and DHA supplementation in the intervention group in another study. The follow-up periods varied strikingly among the various studies, ranging from 6 months to 2 years, and therefore the outcome variables were not sampled at the same time points. The definition of “recent” for recent-onset T1DM was far from similar among studies, ranging from 1 month to 1 year. Some studies focused on immunologic and cytokines markers, while others focused on measures of glycemic control or measures of residual beta-cell function. The effect of patients’ age, gender, latitude, skin pigmentation and race, sun exposure, and season outcome were not systematically recorded and analyzed. Thus, conducting subgroup analyses based on the above-mentioned factors that are critical in any study involving vitamin D was not possible. Such analyses might have provided us with nuanced insights into specific populations that could benefit from vitamin D supplementation. 

## 5. Conclusions and Suggestions for Future Research

From a mechanistic standpoint, theoretically, vitamin D supplementation was hoped to protect from continued beta-cell destruction in patients with new-onset T1DM, through the promotion of helper T-cell subtypes 2 and the repression of helper T-cell subtype 1 cytokines profile [4]. The conclusion of the authors of this systematic review is that at this point in time, there is no strong evidence that vitamin D (or its metabolites) supplementation has any lasting effect on beta-cell preservation or on the glycemic control of newly diagnosed patients with T1DM. However, future research that should be able to prove or disprove our conclusion in a more convincing manner should be conducted in a more homogeneous manner. First of all, studies should be conducted very close to the onset of T1DM, within weeks, and not months to years, when there is very little residual endogenous insulin production to preserve. Second, both clinical outcomes and laboratory outcomes should be better standardized, at pre-defined intervals, both in terms of short-term outcomes (weeks and months) and long-term outcomes (years). The studies should be placebo controlled, and the comparison should not be with another vitamin or another kind of therapy in order to avoid adding too many confounders. Finally, since prevention is always preferable to treatment, it is important to determine in the future whether vitamin D supplementation in patients genetically at risk for the development of T1DM allows for the prevention or delay in the onset of the disease. All such studies should also take into account the role of natural vitamin D obtained from sunlight exposure and diet and how these sources of vitamin D might interact with supplementation. 

Nevertheless, in view of the effects of T1DM on bone mass and the risk of osteopenia [21], it is advisable that all patients with new-onset T1DM should ensure that their vitamin D intake is appropriate and that their vitamin D stores are repleted, regardless of issues of diabetes control or of beta-cell function preservation. 

## Figures and Tables

**Table 1:** Summary of the 11 articles retained in the quantitative analysis.

Author (Year)	Location (Latitude)	Type	Daily Dose	Control (P/N/D)	Blinding	Ages Range	Age (Median)	Time from Diagnosis (mo)	Enrollment Period	FU (Year)	Sample Size	
											Treatment	Control
Pitocco (2006) [9]	Rome, Italy (41.9 N)	1,25	0.25 mcg qod	N	0	>5	13.6	1	*	1	34	33
Walter (2010) [10]	Bavaria, Germany (48.8 N)	1,25	0.25 mcg	P	1	18 to 39	*	2	November 2000–November 2006	1.5	25	18
Bizzarri (2010) [11]	Rome, Italy (41.9 N)	1,25	0.25 mcg	P	1	11 to 35	18	3	*	2	15	12
Gabbay (2012) [13]	San Paulo, Brazil (23.55 S)	D3	2000	P	1	7 to 30	*	6	10 March 2006–28 October 2010	1.5	17	18
Ataie Jafari (2013) [14]	Teheran, Iran (35.7 N)	1 alpha	0.25 to 0.5 mcg	P	1	8 to 15	10.7	2	September–December 2010	0.5	29	25
Haller (2013) [15]	Gainesville, FL, USA (29.65 N)	D3	2000	DHA	0	1 to 18	7.00	3.8	22 April 2009–31 August 2010	1	10	5
Treiber (2015) [16]	Graz, Austria (47.4 N)	D3	70/kg	P	1	>6, 9.5 to 17.5	12.5	3	*	1	14	15
Shih (2016) [17]	Los Angeles, CA, USA (34.1 N)	D3	2857	P	0	13 to 21	16.7	12	October 2012–April 2013	1	12 (IT)	13 (DT)
Sharma (2017) [18]	Puducherry, India (11.9 N)	D3	2000–4000	Insulin only	1	1 to 18	9.3	4.4	August 2014–2015	0.5	26	26
Kadhim (2018) [19]	Bagdad, Iraq (33.3 N)	D3	2000	Insulin only	0	4 to 12	8.5	12	April 2015–May 2016	0.25	25	25
Nwosu (2022) [20]	Worcester, MA, USA (42.3 N)	D2	7143 then 3571	P	1	10 to 21	13.8	3	19 October 2017–12 April 2021	1	18	18

* = no data provided; 0 = no; 1 = yes; P = placebo; N = nicotinamide; DHA = docosahexaenoic acid; FU = follow-up period; IT = immediate treatment; DT = delayed treatment.

**Table 2:** Outcome variables examined in the various 11 studies.

Year	Author	25OHD	HbA1C	Insulin Dose	FCP	Peak-CP	AUC CP	TNF Alpha	IL-4, IL-1β	IL-6	IL-10	Chemokine IL10	IL-12	CD4/CD8	CRP	TREG
2006	Pitocco [9]	0	1	1	1	1	0	0	0	0	0	0	0	0	0	0
2010	Walter [10]	0	1	0	1	1	1	0	0	0	0	0	0	0	0	0
2010	Bizzarri [11]	0	1	1	1	1	1	0	0	0	0	0	0	0	0	0
2012	Gabbay [13]	1	1	1	1	1	0	1	0	1	1	1	1	0	0	1
2013	Ataie Jafari [14]	0	1	0	1	0	0	0	0	0	0	0	0	0	0	0
2013	Haller [15]	1	1	0	0	1	1	0	0	0	0	0	0	1	0	0
2015	Treiber [16]	0	0	1	1	1	1	0	0	0	0	0	0	1	0	1
2016	Shih [17]	1	1	0	0	0	0	1	0	1	0	0	0	0	1	0
2017	Sharma [18]	1	1	1	0	0	0	0	0	0	0	0	0	0	0	0
2018	Kadhim [19]	0	0	0	0	0	0	0	0	0	0	0	0	0	0	0
2022	Nwosu [20]	1	1	1	1	1	0	1	0	1	1	0	1	0	0	0

0 = no; 1 = yes; 25OHD = 25-hydroxyvitamin D; HbA1C = hemoglobin A1C; FCP = fasting c-peptide; AUC CP = area under the curve C-peptide; CRP = C-reactive protein; TREG = T regulatory cells; IL = interleukin.
